# Nonlinear Imaging using Object-Dependent Illumination

**DOI:** 10.1038/s41598-018-37030-7

**Published:** 2019-01-24

**Authors:** Jen-Tang Lu, Alexandre S. Goy, Jason W. Fleischer

**Affiliations:** 1 Department of Electrical Engineering, Princeton University, Princeton, NJ 08544 USA; 2 MGH & BWH Center for Clinical Data Science, Cambridge, MA 02114 USA; 3 Department of Mechanical Engineering, Massachusetts Institute of Technology, Cambridge, MA 02139 USA

## Abstract

Nonlinear imaging systems can surpass the limits of linear optics, but nearly all rely on physical media and atomic/molecular response to work. These materials are constrained by their physical properties, such as frequency selectivity, environmental sensitivity, time behavior, and fixed nonlinear response. Here, we show that electro-optic spatial light modulators (SLMs) can take the place of traditional nonlinear media, provided that there is a feedback between the shape of the object and the pattern on the modulator. This feedback creates a designer illumination that generalizes the field of adaptive optics to include object-dependent patterns. Unlike physical media, the SLM response can provide a wide range of mathematical functions, operate over broad bandwidths at high speeds, and work equally well at high power and single-photon levels. We demonstrate the method experimentally for both coherent and incoherent light.

## Introduction

Feedback is a natural way to engineer nonlinearity, as it creates a functional relationship between input and output. Commonly used in control systems, feedback can act to amplify^1^, filter^2^, and process signals^3^. Historically, there have been two main applications, with two distinct approaches and methodologies: logical operations, with a focus on small degrees of freedom and stable processing, e.g.^4^ and dynamical systems, with an emphasis on large degrees of freedom and instability, e.g. pattern formation^5^. Here, we combine and extend these approaches to improve information transfer in nonlinear imaging systems.

**Figure 1:**
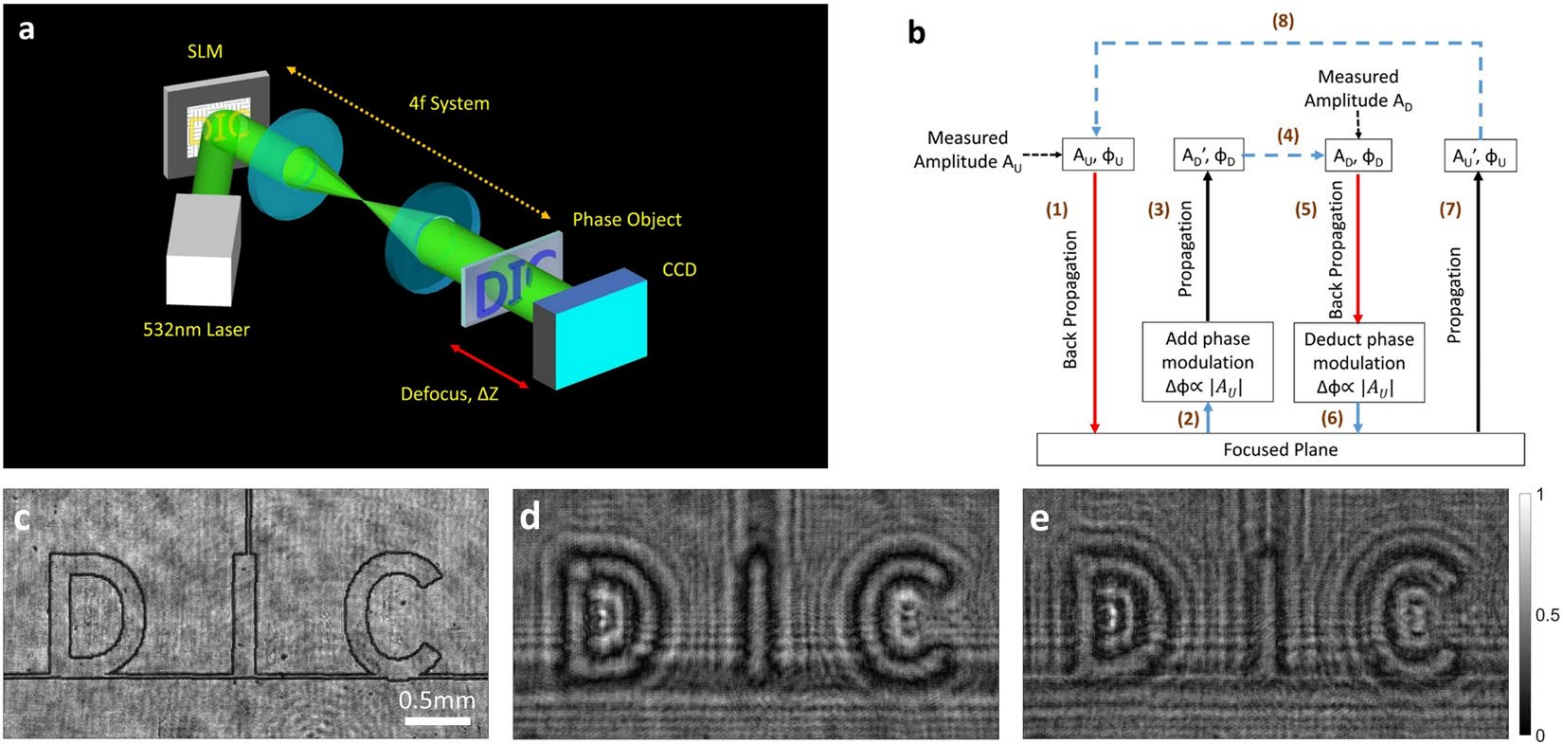
Principle of nonlinear digital imaging. (**a**) Experimental setup. A collimated laser beam is first sent onto a reflective SLM. The surface of the SLM is imaged onto the object via a 4-f lens relay. A CCD camera collects the light transmitted through the sample at a defocused distance ∆z = 4.5 cm. (**b**) Pseudo-code for the image retrieval algorithm (see Methods for details). (**c**–**e**) Experimental intensity measured at (**c**) the focal plane, (**d**) the defocused plane with uniform illumination, and (**e**) the defocused plane with nonlinear (designer) illumination.

The experimental design is shown in Fig. 1a. The method consists of two basic steps. First, a standard image is obtained using uniform illumination. Second, the measured pattern is fed into a spatial light modulator (SLM) and used to re-illuminate the object. The resulting feedback loop between the (digital) image of the object and the (digital) pattern of illumination effectively creates a nonlinearity that can be controlled electro-optically. Unlike nonlinearities that are determined – and limited – by physical media, such as crystals and polymers, SLMs are robust to the environment, respond well to wide ranges of intensity, bandwidth, and coherence, and can generate nearly any mathematical form. This nonlinear tunability (on a pixel-by-pixel basis), both by itself and for optimization on different figures of merit (such as contrast, resolution, edge detection, etc.), distinguishes our approach from earlier attempts at feedback^6^.

Our feedback generalizes two well-known pathways in computational imaging: adaptive optics and structured illumination. In the former, aberrations along an optical path are “pre-compensated” by first measuring and then correcting for the distortions, e.g. using deformable mirror arrays. This has enabled a revolution in remote sensing and ground-based astronomy, e.g. for imaging through a varying atmosphere, and is used routinely in ophthalmology for optical examination of the retina. It is also experiencing resurgence in imaging through turbid media, such as paint and fog^7,8^, in which scatterers randomize the information from object to image. However, the adaptation is independent of the object, used instead for correcting the path along the rest of the optical system.

In structured illumination, a patterned light source adds information at the input of the system, allowing more information about the object to be extracted (deconvolved) at the output. Examples include Zernike masks for phase observation^9^, periodic masks for improved resolution^10,11^ and depth sectioning^12^, Bessel^13^ and Airy^14^ beams for extended depth of focus, and random masks, which enable single-pixel cameras^15^ and coherence engineering^16,17^. As in adaptive optics, though, all these techniques use illumination patterns that are independent of the sample.

The primary reason for sample independence is that the object is typically unknown and a universal form of illumination is desired. This results in a general, shift-invariant transfer function that is suitable for arbitrary objects but is optimized for none of them. The introduction of feature-based illumination, through feedback, will improve information transfer and all metrics of image quality.

## Results

In our geometry, the feedback between source and detector closes the loop in computational imaging. While the functional relationship between the SLM and the object is essentially arbitrary, we consider here the simplest case of an intensity-dependent phase modulation: *ϕ*_*SLM*_ = *γI*^*α*^, where γ is the strength of the modulation and α is its order. This makes the system a physical implementation of the conventional split-step method in beam propagation codes, with one step for diffraction and one step for nonlinear effect, and allows a straightforward comparison with more conventional nonlinearities, e.g. the Kerr response for which α = 1. We emphasize, though, that much more complex responses are possible, including the ability to impose separate amplitude and phase modulations using a second SLM. More details about the digital nonlinear response are given in the Supplementary Information.

### Nonlinear phase reconstruction

While the method can be applied to any type of image (provided there is a signal to bootstrap), phase reconstruction is particularly important. Phase contains most of the information in the signal, is crucial for a variety of important applications, and responds particularly well to nonlinearity^18^. Consequently, we experimentally demonstrate the method by nonlinearly improving the imaging of a phase object (Fig. 1c).

We use a modified version of the Gerchberg-Saxton (GS) method^19^ to retrieve the phase, taking advantage of the fact that phase matching from the nonlinear illumination-object feedback provides a strict constraint on the behavior of the algorithm^20^. As the shape of the object is not known *a priori*, we first record a defocused intensity (I_U_ = |A_U_|^2^) that is obtained using uniform illumination (Fig. 1d). The amplitude distribution, |A_U_|, is then fed into a SLM as a phase modulation *ϕ*_*SLM*_∝|*A*_*U*_|, enabling a second intensity measurement (I_D_ = |A_D_|^2^) with designer illumination (Fig. 1e). As in previous examples of nonlinear imaging^18,21^, we use a self-defocusing nonlinearity; this enables sufficient wave mixing to enhance imaging while suppressing the fast instabilities common to self-focusing systems.

From the two images I_U_ and I_D_, the nonlinear phase retrieval algorithm proceeds in a manner similar to GS reconstruction. First, a field with amplitude A_U_ and initial uniform phase *ϕ* = 0 is numerically back-propagated from the (out-of-focus) camera plane to the sample plane (in focus). The phase modulation *ϕ*_*SLM*_ is then added to the phase of the field in order to simulate the action of the SLM, and the new field is forward-propagated to the camera plane. This simulated amplitude is then replaced by the amplitude of the actual measurement (A_D_), and the field is backward-propagated to the sample plane again. After subtraction of the phase modulation ϕ_SLM_ from the phase of the field at the sample plane, the new field is once again forward-propagated to the camera plane, followed by replacement of the numerical amplitude by the measured amplitude A_U._ The process is repeated until convergence. A pseudocode for this algorithm is shown in Fig. 1b.

As a phase object, we used a sample consisting of a glass plate with the three characters “DIC” etched on it. The in-focus image of the sample is shown in Fig. 1c. As the object is thin and transparent, no intensity modulation appear on the image in focus apart from scattering from the character edges. In Fig. 1d,e, we show the measured diffraction intensity distributions in the detector plane (out-of-focus) with uniform and designer (feedback) illumination, respectively. In general, more diffraction fringes are visible in the latter image, since the illumination has the shape of the object itself. The second pass of illumination effectively doubles the diffraction pattern, giving at least a two-fold increase in phase sensitivity to the object. Larger multiplication is of course possible^21^, especially with cascaded interactions^22^, with potentially exponential improvement upon repeated iteration.

**Figure 2:**
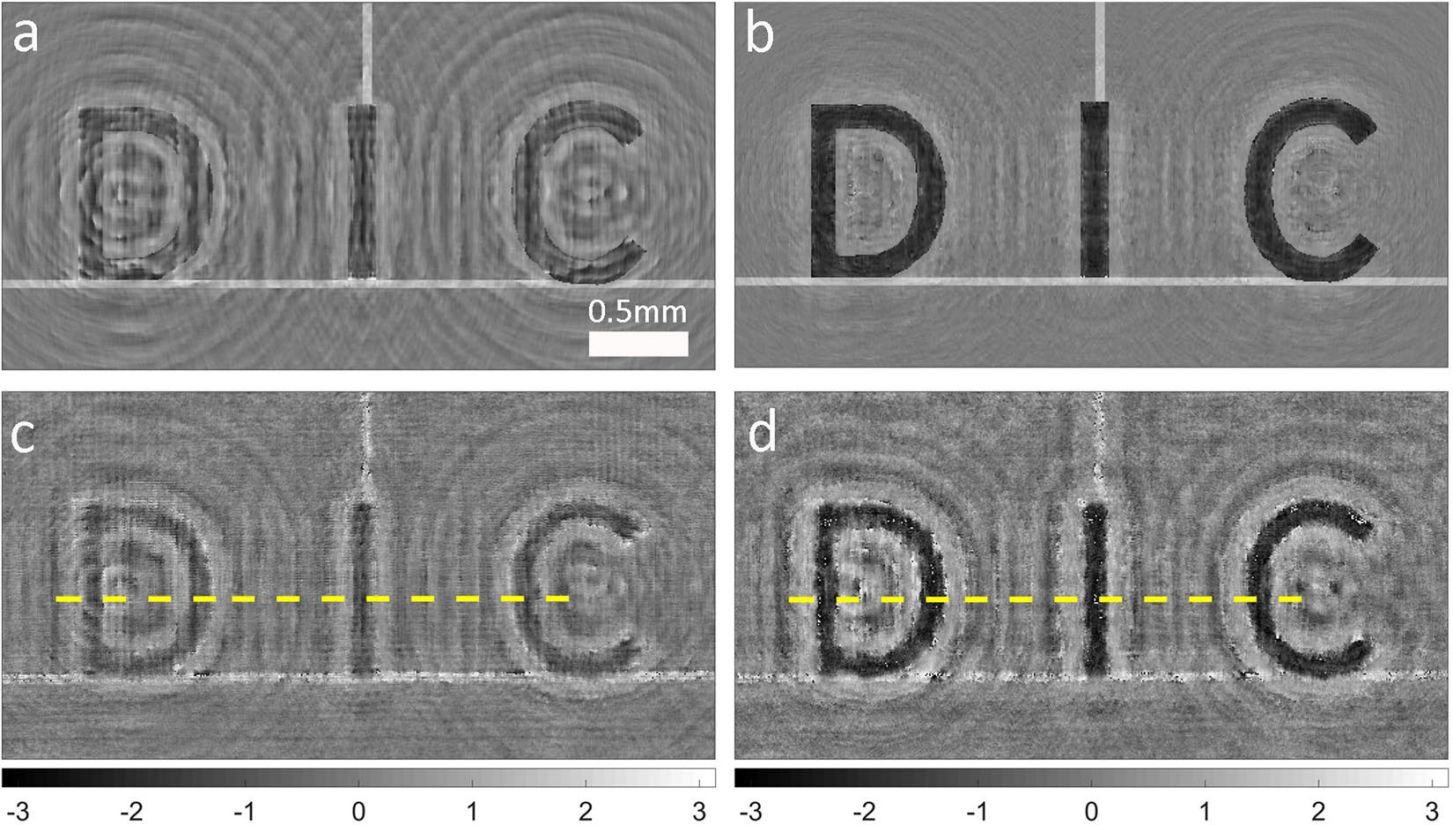
Comparison between linear and nonlinear reconstructions. (**a**,**b**) Simulation of phase reconstruction with (**a**) the linear algorithm and (**b**) the nonlinear algorithm. (**c**,**d**) Experimental phase reconstruction with (**c**) the linear algorithm and (**d**) the nonlinear algorithm using partially coherent light (corresponding to a speckle spatial frequency of 2.5 × 10^4^ rad/m).

We compare our nonlinear algorithm with a variation of the GS algorithm for linear propagation, for the same defocused distance. The conventional algorithm relies on one in-focus and one out-of-focus intensity measurement using uniform illumination. Simulation results are shown in Fig. 2a,b. In real imaging cases, the target is unknown and the reconstructed phase error cannot be accessed; in the simulation test case, the ground truth is chosen *a priori*, and we can quantitatively measure the reconstructed phase error

1 $${\rm{ER}}=\frac{{\sum }_{r{\epsilon }S}|f(r)-g(r)|}{{\sum }_{r{\epsilon }S}|g(r)|}\,$$

where f(r), g(r), and S are the reconstructed image, the ground truth, and the image space, respectively.

As expected, the nonlinear reconstruction (Fig. 2b) is about 2x better than the linear reconstruction (Fig. 2a) in term of the phase error (ER 0.8 vs. 1.5). The experimental counterparts, shown in Fig. 2c,d, confirm the result that the nonlinear algorithm provides much better reconstruction than the linear algorithm. For quantitative comparison, we define contrast and resolution as:

2 $$Contrast={\bar{I}}_{DIC}-{\bar{I}}_{BKG}$$

3 $$R={({\nabla }_{x}I)}_{x\in DICE}^{-1}$$

where $\bar{I}$ represents the mean value of the intensity, the subscript “DIC” denotes the region of the DIC characters, “BKG” denotes the rest of the image, and “DICE” denotes the edge domain of the DIC region. (For a consistent measure of resolution based on a single image, we take the highest resolvable spatial frequency, given by the inverse of the normalized image gradient averaged over all the character edges.) By these metrics, the nonlinear algorithm yields a 260% improvement in contrast (1.50 vs. 0.58) and a 15% improvement in resolution (14.8 vs 17.1 microns) over linear reconstruction.

**Figure 3:**
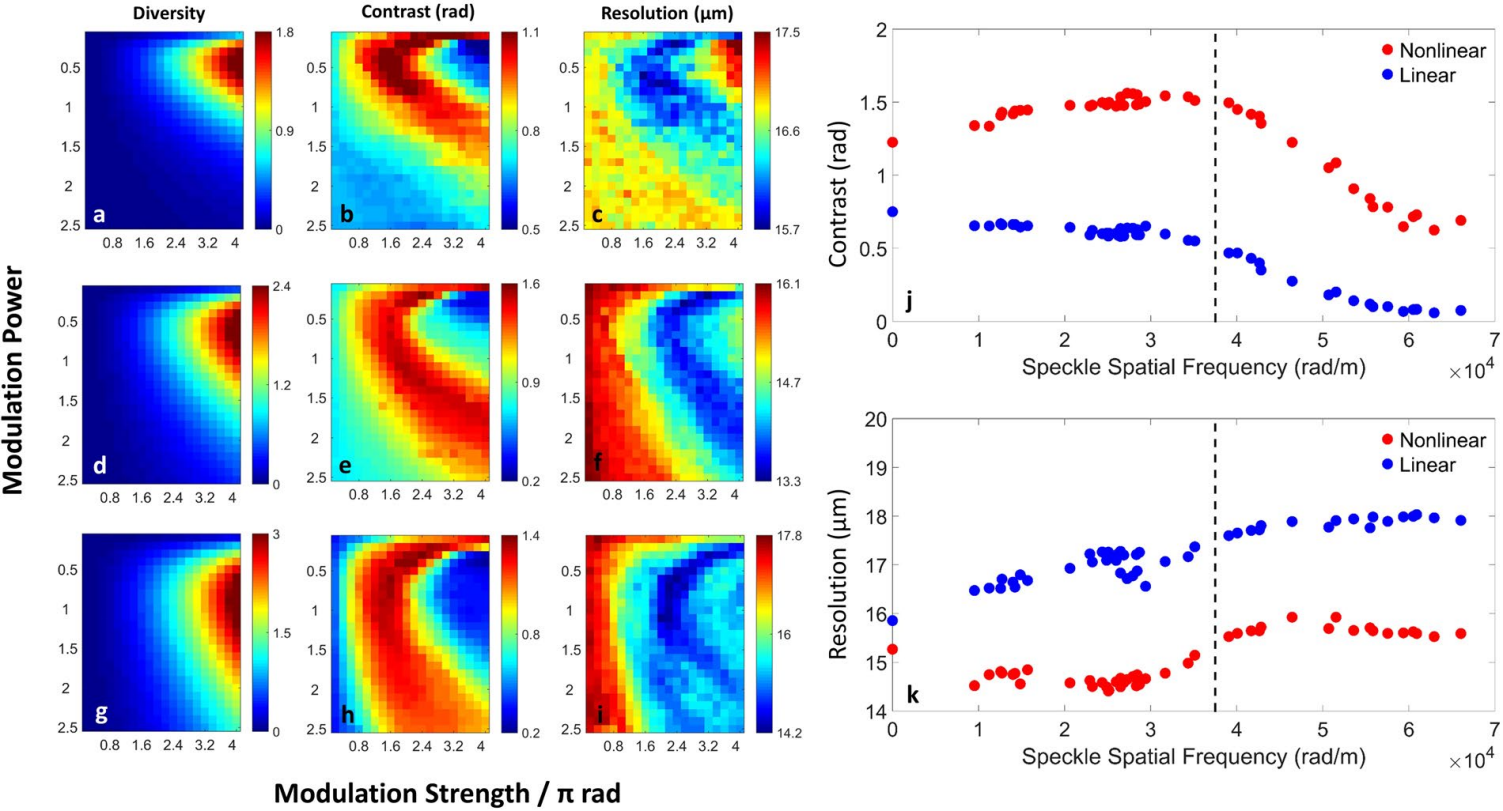
Experimental results as a function of nonlinear strength and power. (**a**–**i**) Show the phase variance (**a**,**d**,**g**), contrast (**b**,**e**,**h**) and resolution (**c**,**f**,**i**) of the nonlinear reconstructions. (**a–c**) Show the nonlinear reconstructions for coherent illumination while (**d**–**f**) and (**g–i**) are reconstructions for partially coherent illumination with speckle spatial frequency 2.3 × 10^4^ rad/m and 4.9 × 10^4^ rad/m, respectively. (**j**,**k**) Comparison of linear (blue) and nonlinear (red) reconstructions for (**j**) contrast and (**k**) resolution. Full coherence is given by zero spatial frequency, and the nonlinearity is fixed at modulation power = 0.5 and modulation strength = 1.5π. The dashed line corresponds to the spatial frequency (3.75 × 10^4^ rad/m), at which the illumination speckle size becomes comparable to the DIC character stroke width. When the spatial frequency exceeds this value, the image quality becomes worse as the statistics washes out the diffraction fringes.

Another advantage of the digital system, and the corresponding algorithm, is the ability to work for different degrees of spatial coherence. We demonstrate this experimentally by making the illumination beam partially spatially coherent by passing it through a rotating diffuser^23^. The performance of linear and nonlinear modulation, in terms of contrast and resolution, are shown in Fig. 3j,k, respectively. In all cases, the figures of merit for linear propagation get worse monotonically as the degree of coherence is decreased (consistent with a progressive decrease in visibility of diffraction fringes^24^). In the nonlinear case, both contrast and resolution are improved simultaneously beyond their linear limits. [This win-win situation, normally an engineering trade-off in linear systems^25^, is a general feature of nonlinear imaging systems^21^.] For designer illumination, the contrast actually improves with decreasing coherence, up to an optimal value, while the system resolution is relatively constant above and below this value. The former is a stochastic resonance effect^26^, peaking when the smallest significant feature of the object (the stroke width of the DIC characters, ~167 μm) matches the spatial correlation length of the illumination (characterized by the speckle spatial frequency 3.75 × 10^4^ rad/m). The latter results from improved visibility due to mean-field (DC) scattering^21^.

## Discussion

Theoretically, the improvements in image quality are unlimited^11,21^. In practice, there are many factors that hinder the performance. These include the usual trade-off between pixel size and dynamic range in the camera and SLM^27^, pixel-mapping and tone-mapping mismatches between devices, amplitude corruption in the phase modulation, and genuine issues of noise. There are also frame-rate limitations if multiple iterations are desired, and issues of convergence and computational complexity as a function of basis, method of phase retrieval^28^, type of nonlinearity, and desired spatial detail (e.g. different class of response for individual SLM pixels).

**Figure 4:**
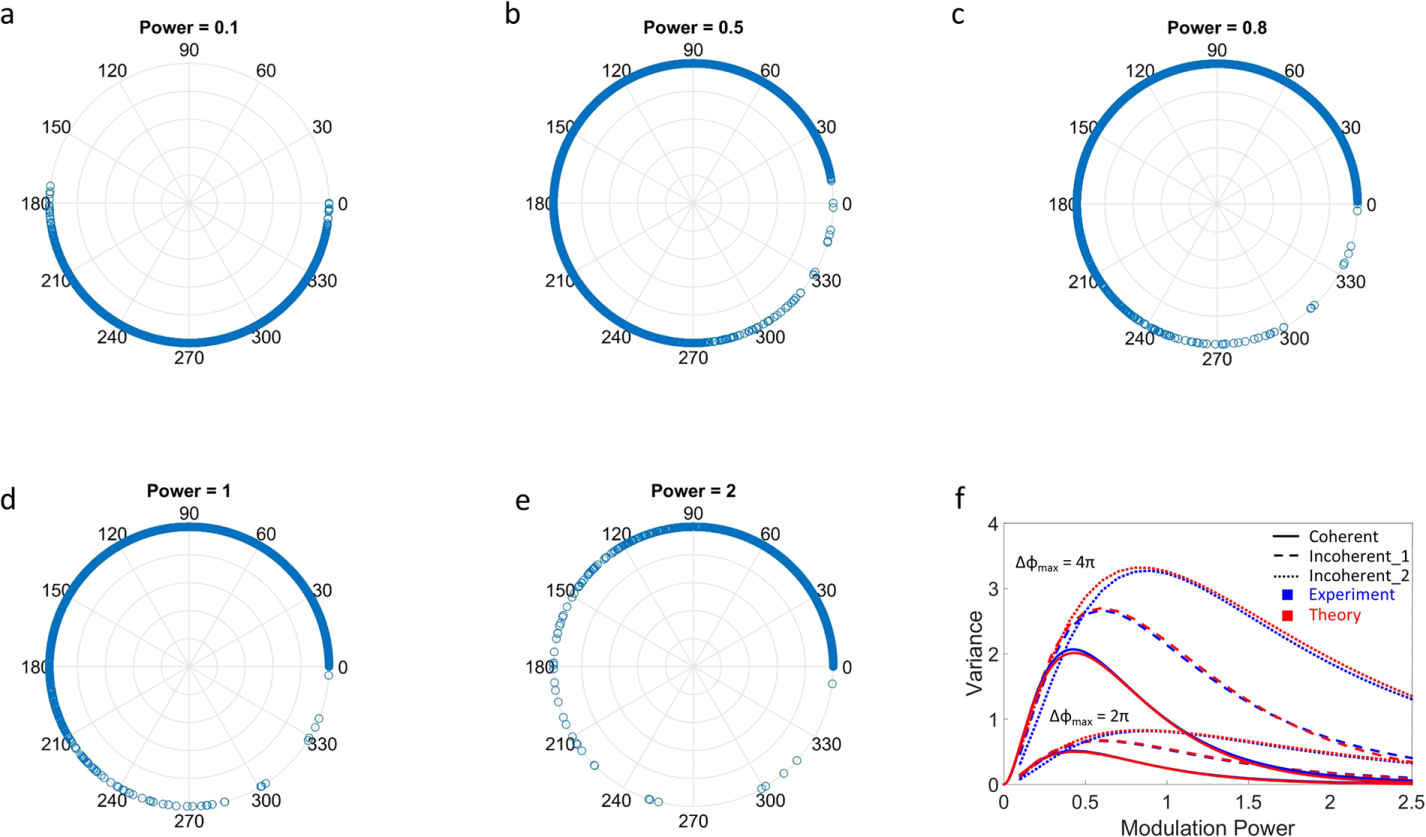
Diversity of phase modulation. (**a**–**e**) Modulation *ϕ*_*SLM*_ = *γI*^α^ for coherent light (strength γ = 2π). The largest phase/information diversity (for better sampling) occurs when α = 0.5. (**f**) Shows the relation between diversity/variance and modulation power. Lower and upper curves represent modulation strengths γ = 2π and 4π, while solid, dashed, and dotted lines denote coherent and partially coherent illumination with speckle spatial frequency 2.3 × 10^4^ rad/m (Incoherent_1) and 4.9 × 10^4^ rad/m (Incoherent_2). Blue: experiment; red: theoretical fits for stretched exponential ${\sigma }_{SLM}^{2}=A{\alpha }^{2}\exp (-B{\alpha }^{C})$, with A = 33 and 132 for the lower and upper curves, B = {4.9, 4.1, 3.7}, and C = {0.8, 0.7, 0.6} for the coherent and incoherent cases.

The best approach to retrieving (estimating) the phase object is to maximize the information in the encoded illumination. This is given by the most widespread sampling in phase space, which for the experimental setup here corresponds to the most diverse distribution of phase on the modulator (Fig. 4). Using variance as a measure, we find that ${\sigma }_{SLM}^{2}=E[{(X-\mu )}^{2}]=A{\alpha }^{2}\exp (-B{\alpha }^{C})$, where X represents the pixel value on the SLM, μ = E[X] is the mean value of all the pixels, and α is the modulation power. This stretched-exponential form suggests a nontrivial (i.e. non-diffusive) optical flow as the object modes are mixed by the modulator^26,29–31^. As shown in Fig. 3a–i, the optimal system response ranges from a strict proportionality to amplitude $({\varphi }_{SLM}=\gamma \sqrt{{I}_{U}})$ for the coherent case to a more conventional Kerr nonlinearity $({\varphi }_{SLM}=\gamma {I}_{U})$ for the incoherent case.

These optimum transfer functions can be understood by considering the extremes of nonlinear response. For very weak response, there is little difference between linear and nonlinear output, and therefore little gain in information. Stronger responses give more significant differences, evidenced by more pronounced intensity fringes as modes interfere, until high-intensity regions start dominating the image. Above this point, the growth of hot spots leads to a modulation that is not indicative of the object as a whole (an effect exacerbated by noise, e.g. through modulation instability). From an information perspective, the structured feedback acts as a nonlinear matched filter^32^, with an amplitude-dependent response appropriate for the optical transfer function of coherent light and an intensity-dependent response appropriate for the modulation transfer function of incoherent light^33^. More details of this response are given in the Supplemental Information.

The quality of the reconstructed image using the nonlinear algorithm is comparable to what we obtained in a previous work using a nonlinear photorefractive crystal^18^. In that system, the intensity pattern of the object induced an index change in the crystal, creating a self-reinforcing diffraction grating during light propagation. Here, the object-dependent pattern on the spatial light modulator creates a similar grating (computer-generated hologram) in the plane of the SLM, which acts more like an iterative map. In both cases, the nonlinear feedback means that the grating structure is automatically phase-matched with the object, guaranteeing an optimized diffraction pattern. For the computational part, the interplay between intensity and phase gives an extra constraint on the algorithm, resulting in improved phase sensitivity, selectivity, and convergence.

While electro-optic devices have their limitations, they are far more versatile than the physical media traditionally used for nonlinear optics. Of particular significance is the ability to create nonlinearities that are distinct from those of physical media by leveraging independent control of amplitude and phase modulation. This freedom opens new applications for functional forms that have remained purely in the mathematical domain. The ability to adjust parameters dynamically also holds much promise for improving the efficiency and performance of adaptive optics and compressed sensing/imaging. Finally, digital methods have the potential to revolutionize imaging at extremely low light levels, such as fluorescence microscopy and quantum imaging, as SLMs are inherently non-destructive and can operate at the single photon level.

## Methods

A 532 nm Coherent Verdi laser was used as a light source, and a phase-only spatial light modulator (SLM, Holoeye PLUTO, with a resolution of 1920 × 1080 pixels, 8-micron pixel size, and 8-bit depth) was placed in the illumination path before a phase object, modulating the illumination light as a function of the diffracted pattern of the sample. As a direct image does not reveal the object, we defocused the CCD camera (with a resolution of 1280 × 1024 pixels, 6.7-micron pixel size, and 16-bit depth) a distance Δz = 4.5 cm from the image plane and recorded the subsequent diffraction pattern. To produce partially incoherent light, the experimental setup was modified by inserting a rotating diffuser in the illumination path, and the degree of spatial incoherence was quantified by measuring the spatial frequency of the speckles, *i*.*e*. 2π over the correlation length of speckles at the detection plane.

To align the SLM and the CCD camera for feedback, it is essential to find the transformation matrix between the two devices. A 100 × 100 square mask was created on the SLM and moved in x and y direction with an interval of 200 pixels; we then found 25 point-by-point correspondences between the SLM and the camera by measuring the centroids of the mask on the SLM and the corresponding pattern on the camera. A 3 × 3 transformation matrix between the two devices was thus calculated.

From the uniform- and designer-illuminated images I_U_ and I_D_, the nonlinear phase retrieval algorithm proceeds as follows:

1. An object field u_rec_ with amplitude A_U_ and initial uniform phase 𝜙 = 0 is numerically back-propagated from the (out-of-focus) camera plane to the (in-focus) sample plane.
2. A phase modulation 𝜙_*SLM*_ is added to the phase of u_rec_ to simulate the action of the SLM.
3. The new field is forward-propagated to the camera plane, yielding a field u′_D_ with amplitude A′_D_.
4. The simulated amplitude A′_D_ is replaced by the amplitude of the actual measurement A_D_.
5. The field is back-propagated again to the sample plane, yielding a new field u_rec_.
6. The phase modulation 𝜙_*SLM*_ is subtracted from the phase of u_rec_.
7. The field is forward-propagated, yielding an estimate of the un-modulated image amplitude A′_U_.
8. A new field is generated by replacing A′_U_ with the actual measurement A_U_. At this point, the algorithm restarts from step 1.

This algorithm effectively interpolates between the infinitesimal displacement of transport-of-intensity algorithms^34^ and the infinite (far-field) displacement of the Gerchberg-Saxton algorithm^19^. That is, it works for any distance of defocus, subject to the limits of Fresnel diffraction (which can be modified by changing the propagator). The method also works for arbitrary degrees of spatial coherence, as in this case the algorithm converges on an overall envelope phase^35^.

## Supplementary information

Supplementary information
