# Supplementary material for: Nonlinear Imaging using Object-Dependent Illumination
Source: Sci Rep. 2019 Jan 24;9:725. doi: 10.1038/s41598-018-37030-7 (PMC6345888; doi:10.1038/s41598-018-37030-7)
Supplement: Supplementary file 1 — Supplementary information [file 41598_2018_37030_MOESM1_ESM.pdf]

Supplementary Information for

Nonlinear imaging using object-dependent illumination

Jen-Tang Lu, Alexandre S. Goy, and Jason W. Fleischer*

*E-mail: jasonf@princeton.edu

This PDF file includes:

Supplementary Text
Supplementary Figures

Supplementary Text:

This Supplementary Information gives more details about the digital nonlinear response. It gives an experimental demonstration of digital modulation instability with a phase-only modulator and discusses issues of information diversity and noise.

1. Modulation instability (MI)

Perhaps the simplest nonlinear effect to consider is modulation instability, since its dynamics can be demonstrated easily with a uniform plane wave (matching the uniform illumination used in the experiment). In MI, amplitude perturbations can grow in a self-focusing medium and damp in a self-defocusing one, as intensity-dependent changes in the refractive index create local converging and diverging lens profiles, respectively.

There are two interesting features when modeling MI. The standard theoretical analysis, using linearized perturbation theory, gives a growth rate that is independent of the amplitude of the perturbation. Rather, it represents a competition between nonlinear self-focusing and diffraction, with the former determined by the intensity of the background. In numerical simulation, e.g. using a split-step beam propagation code, the nonlinearity gives a phase change (through the change in refractive index) but the perturbation amplitude in the simulation is retained. Indeed, for propagation (vs. initial growth), it is this amplitude/intensity which is fed back into the code for further evolution of the wave.

As mentioned in the text, the method of digital nonlinear imaging is effectively a physical implementation of the split-step method. For full-field feedback, then, the dynamics should be exactly the same as traditional beam propagation. For phase-only feedback, the amplitude from

the SLM is reset to uniform intensity at the beginning of every iteration. Nevertheless, unstable dynamics is preserved, as it is the phase profile (curvature) that determines the evolution of the wave. Indeed, we show in Supplementary Figure 1 that the current experimental method reproduces the appropriate features of MI, viz. growth for self-focusing and damping for self-defocusing nonlinearity.

2. Nonlinear response

Even in the ideal case, with no noise, there is an optimal value of nonlinearity for a given imaging system. For very weak response, there is little difference between linear and nonlinear output, and therefore little gain in information. Stronger responses give more significant differences, evidenced by more pronounced intensity fringes as modes interfere, until high-intensity regions start dominating the image. Above this point, the growth of hot spots leads to a modulation that is not indicative of the object as a whole (an effect exacerbated by noise, e.g. through MI). Examples of this sequence, for coherent and partially coherent light, are shown in Supplementary Figures 2 and 3.

As discussed in the text, the optimal system response is given by the most diverse distribution of phase on the modulator. This corresponds to the most robust fringes on the feedback pattern (Supplementary Figure 3a and Supplementary Figure 3f).

3. Limitations

In practice, the possible nonlinear improvement in image quality is capped by limitations in the camera and/or SLM. Of these, the finite dynamic range is the most significant, as new modes generated by the nonlinearity may be weaker than the noise/intensity floor that can be detected. A numerical simulation of this is shown in Supplementary Figure 4.

We note that in the typical spirit of computational optics, many of the single-shot limits in resolution, contrast, and dynamic range can be overcome by taking multiple images over a broader range of scales.

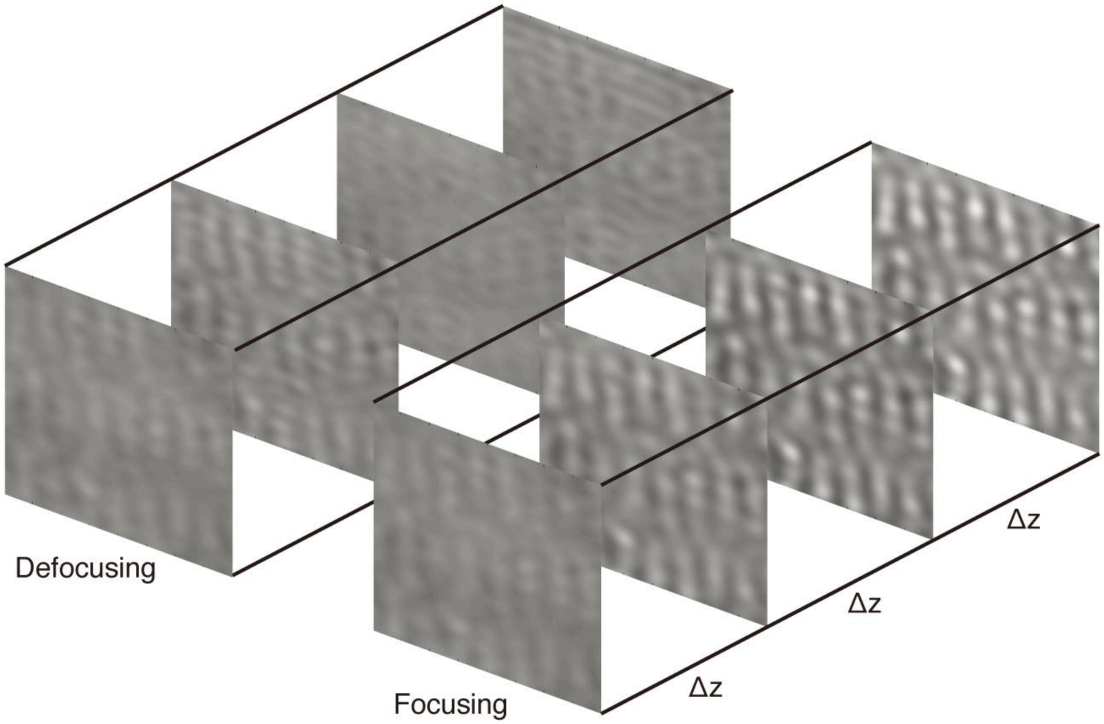

Supplementary Figure 1 | Experimental demonstration of modulation instability (MI). Shown are CCD images after repeated application of a phase-only digital nonlinearity for an initial sinusoidal phase perturbation. As in the usual full-field propagation of MI, the self-focusing case is unstable and the self-defocusing case is stable.

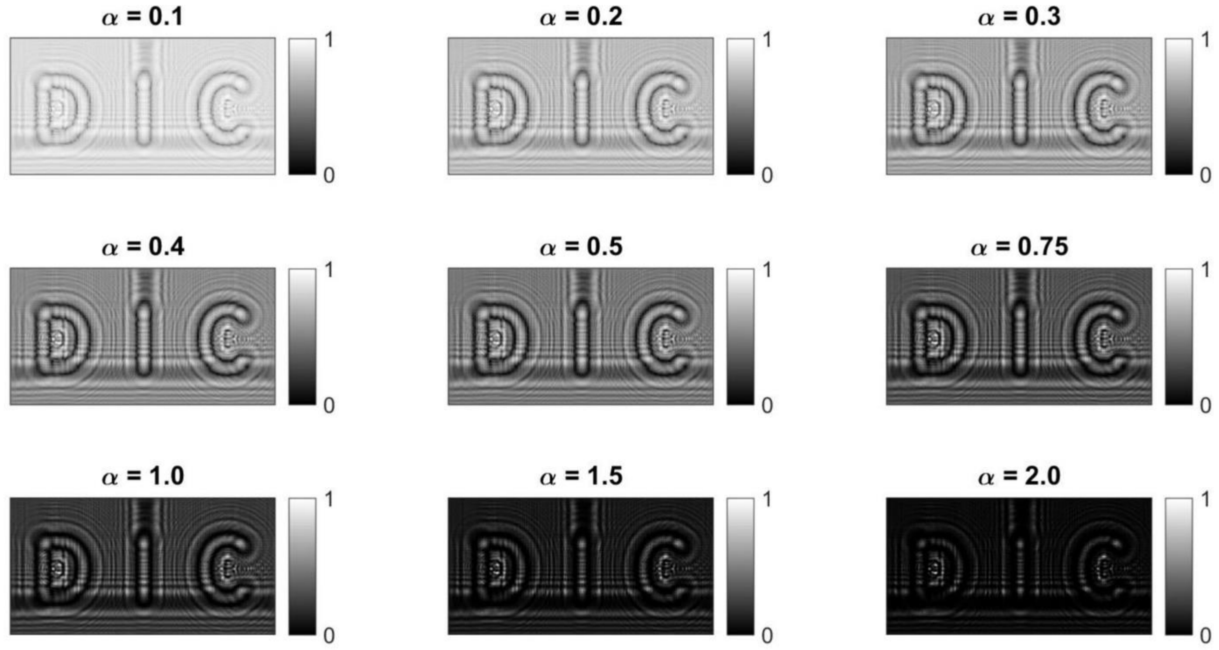

Supplementary Figure 2 | Simulated behavior for coherent light with nonlinear response I_U^α. There is an optimal response for α = 0.5. For smaller exponents, the nonlinear effect is weak and diffraction dominates. For larger exponents, too much wave mixing occurs and high-intensity spots dominate the output.

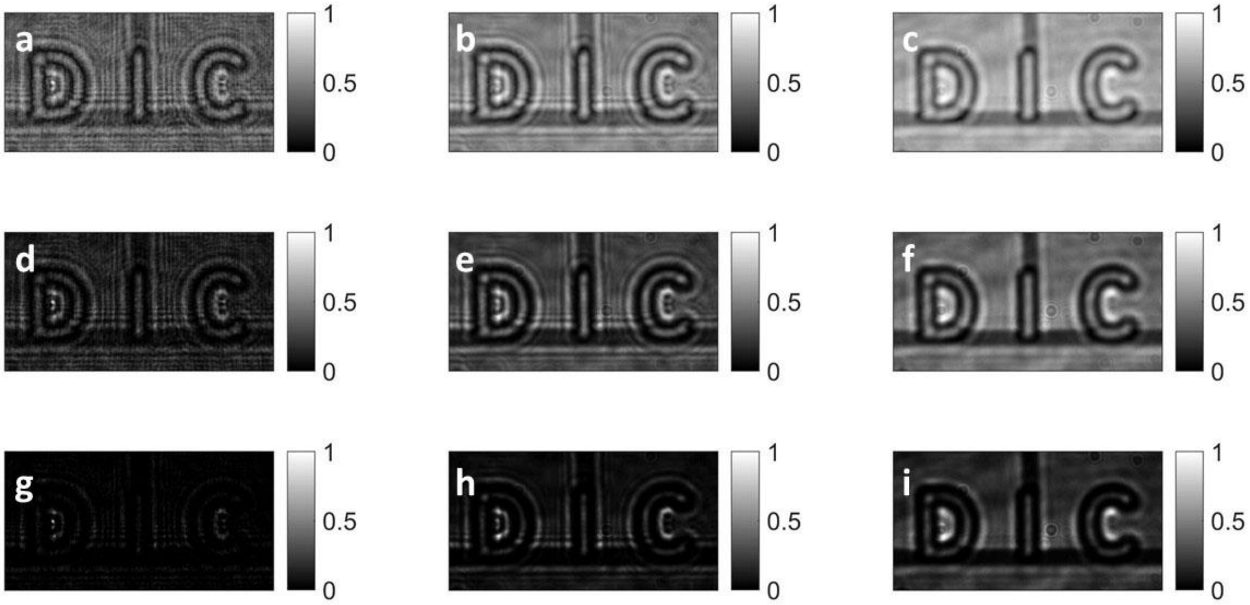

Supplementary Figure 3 | Experimental measurements of intensity for different power of modulator response. Distributions of $I_U^{0.5}$ using (**a**) coherent light, and (**b,c**) partially coherent with speckle spatial frequency (**b**) 2.3×10^4 rad/m and (**c**) 4.9×10^4 rad/m. (**d-f**) Distributions of I_U corresponding to the same spatial coherence of (a-c). (**g-i**) Distributions of I_U^2 corresponding to the same spatial coherence of (a-c).

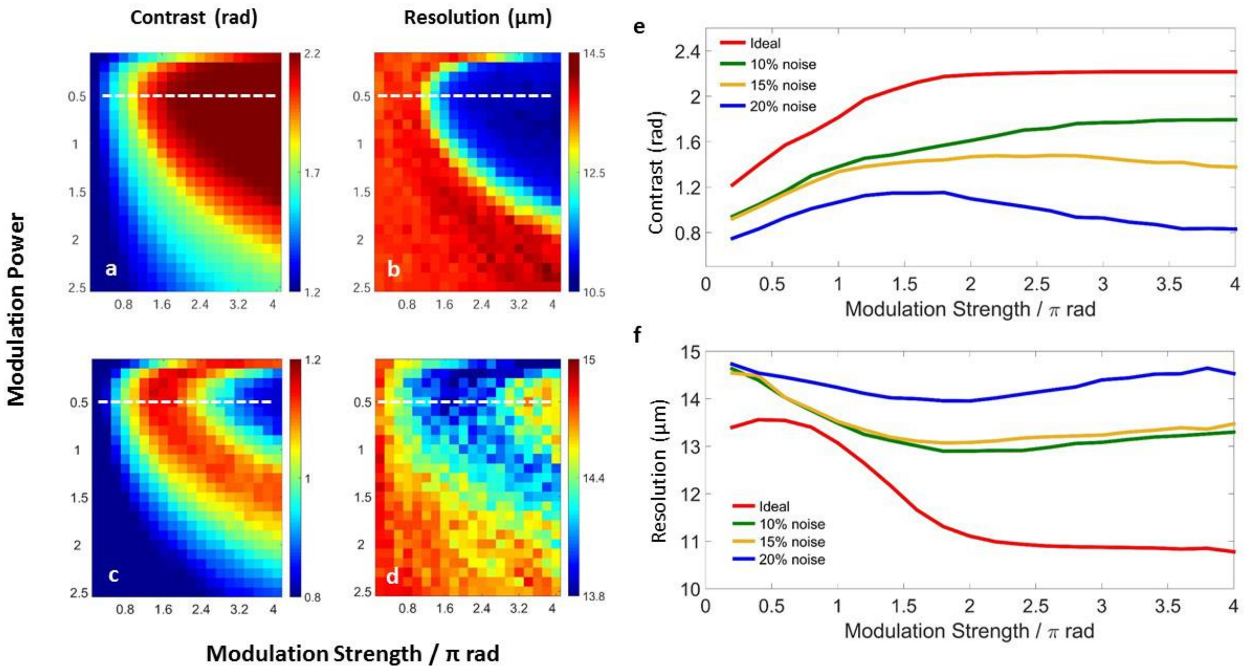

Supplementary Figure 4 | Simulation results of system performance with and without noise. **a-d**, Contrast and resolution (**a,b**) without and (**c,d**) with noise. **e,f,** Cross-sections of (a-d) showing competition between nonlinear modulation strength and noise.
